# Maternal smoking during pregnancy and risk of phacomatoses: results from a Swedish register-based study

**DOI:** 10.2147/CLEP.S216634

**Published:** 2019-09-03

**Authors:** Giorgio Tettamanti, Hanna Mogensen, Ann Nordgren, Maria Feychting

**Affiliations:** 1 Institute of Environmental Medicine, Unit of Epidemiology, Karolinska Institutet, Stockholm, Sweden; 2 Department of Molecular Medicine and Surgery, Center for Molecular Medicine, Karolinska Institutet, Stockholm, Sweden; 3 Diagnostic Services, Karolinska University Hospital, Clinical Genetics, Stockholm, Sweden

**Keywords:** phacomatoses, neurofibromatoses, smoking, pregnancy, epidemiology

## Abstract

**Background and aim:**

Phacomatoses are genetic syndromes often associated with an increased risk of a variety of malignant and benign neoplasms, including nervous system tumors. Little is known about the causes of de novo occurrences of phacomatoses. Therefore, the aim of this study was to assess the association between maternal smoking during pregnancy and the occurrence of de novo phacomatoses.

**Methods:**

All individuals born in Sweden between 1982 and 2014 with information on both biological parents were identified through the Medical Birth Register (MBR), n=3,132,056. The Swedish population-based health care registers were used to identify individuals with a phacomatosis and information on maternal smoking was extracted from the MBR. Logistic regression models were used to evaluate the effect of maternal smoking during pregnancy on the risk of phacomatoses.

**Results:**

In the study population, we identified 2074 individuals diagnosed with a phacomatosis, among which 75% were regarded as de novo occurrence. While no effect of heavy maternal smoking (10+ cigarettes/day) was observed for de novo neurofibromatosis, an increased risk was found for other phacomatoses excluding neurofibromatosis (OR =1.51, 95% CI 1.13–2.03). Indications of an increased risk for specific phacomatosis subtypes were observed for tuberous sclerosis (OR =1.39, 95% CI 0.91–2.14) and Sturge–Weber syndrome (OR =1.86, 95% CI 0.83–4.19). No association was observed for familial phacomatoses.

**Conclusion:**

This is the first study examining the risk of de novo phacomatoses associated with heavy maternal smoking during pregnancy. Further studies are needed to confirm the associations observed and elucidate potential biological mechanisms.

## Introduction

Phacomatoses are genetic, neurocutaneous syndromes, manifesting at different ages with central nervous system and cutaneous abnormalities.^1^ Moreover, these syndromes are associated with an increased cancer risk and most are characterized by multiple hamartomas of the central and peripheral nervous system, eye, skin, and viscera.^2^,^3^ Several phacomatoses are known to be associated with the occurrence of nervous system tumors.^4^,^5^ The etiology behind phacomatoses is very heterogeneous and the different disorders are caused by a large number of genetic mechanisms, including mosaicism, point mutations, and single gene disorders with different inheritance patterns. Many of these disorders have an autosomal dominant inheritance pattern and are associated with a range of mild to severe developmental disabilities.^1^ The most common phacomatoses are neurofibromatosis type 1 and type 2, tuberous sclerosis, and Sturge–Weber syndrome: these syndromes are relatively rare and have a prevalence at birth of approximately 1 in 3000, 1 in 35,000, 1 in 10,000, and between 1 in 20,000–50,000, respectively.^1^,^6^–^8^

Although phacomatoses are mainly autosomal dominant disorders, a large proportion of the cases has no family history of the disease: the majority of tuberous sclerosis cases (between 65% and 85%) stems from de novo mutations, as well as approximately 50–75% of the neurofibromatosis cases.^7^,^9^ Sturge–Weber syndrome is not inherited but occurs as a somatic mosaic mutation during embryonic development.^10^ Due to the large proportion of de novo phacomatoses, it is important to identify risk factors for these disorders. Parental characteristics and exposures that occurred around the time of conception may be related to the risk of specific de novo phacomatoses, but very few studies are available. Thus, there is currently very little knowledge about potential risk factors; however, recent studies have reported an increased risk of de novo phacomatoses, particularly neurofibromatosis, associated with advanced paternal age, while no effect was observed for maternal age.^11^–^13^

Maternal smoking during pregnancy is a risk factor for many birth outcomes, such as low birth weight, preterm birth, stillbirth, and birth defects.^14^,^15^ Regarding its effect on specific genetic syndromes little is known. In studies of Down syndrome, the results are not conclusive. In some, mainly small studies maternal smoking was associated with a decreased risk of Down syndrome^16^–^18^ but larger studies did not confirm this finding.^19^,^20^ However, a study that categorized Down syndrome cases according to the parental origin of the extra chromosome 21 and timing of the meiotic error, reported that maternal smoking during pregnancy increased the risk of a subset of Down syndrome, caused by errors that occurred in maternal meiosis II.^21^

The aim of the current study was to evaluate the effect of maternal smoking during pregnancy on the risk of de novo phacomatoses in offspring as a whole group and, when possible, for specific diagnoses, using data from the nationwide Swedish Medical Birth Register (MBR) and population-based health care registers.

## Methods

All individuals born in Sweden between 1982 and 2014, registered in the MBR and with information on maternal smoking during pregnancy were eligible for inclusion into the study. The MBR was established in Sweden in 1973 and contains information about antenatal, obstetrical, and neonatal care: it has been estimated that approximately 98% of the children born in Sweden are included in the MBR.^22^ Information regarding maternal smoking during the first trimester is available from 1982 onwards and is recorded in the MBR as: non-smoker, 1–9 cigarettes/day (light smoking), and 10+ cigarettes/day (heavy smoking). We used non-smoker as reference category. The Multi-Generation Register was used to identify the biological parents of all the eligible individuals. The Multi-Generation Register contains information regarding biological (and adoptive) parents for individuals born 1932 or later that has been registered in Sweden any time since 1961.^23^ For children born in Sweden 1961 or later, the coverage is very high and information on the biological mother and father is available for 100% and 98%, respectively.23 For the current study, 3,388,508 individuals were identified from the MBR, and among them more than 93% had valid information regarding maternal smoking during pregnancy (n=3 157,922). Individuals who did not have information on both biological parents were excluded (n=25,866, <1%), which left 3,132,056 individuals in the study population.

The Inpatient and Outpatient Registers, the MBR, and the Cause of Death Register were used to identify individuals diagnosed with a phacomatosis. A phacomatosis is present at birth, but may not be detected until later in life. Therefore, for complete case ascertainment, it is essential to be able to follow the entire study population in health data registers over an extended period of time. The Inpatient Register records the primary and secondary (currently up to 30) diagnoses for all hospitalizations. It was established in 1964, and became nationwide in 1987.^24^ From 2001, diagnoses in outpatient specialist care are recorded in the Outpatient Register. The Cause of Death Register was established in 1952.^25^ During the study period, diagnoses in these register have been coded according to the 8th, 9th, and 10th revisions of the International Classification of Disease (ICD) (Table S1).

In order to distinguish between de novo and familial phacomatosis cases, the Multi-Generation Register was used to identify biological parents, siblings (full and half), grandparents, uncles, aunts, nephews, and cousins for all individuals diagnosed with a phacomatosis: if at least one identified relative also had a diagnosis of a phacomatosis in the Inpatient or Outpatient Registers, the case was defined as familial otherwise it was regarded as de novo. The most recent diagnosis was used to define the phacomatosis subtype. All register data used for the current study are held at the National Board of Health and Welfare (Socialstyrelsen).

The effect of maternal smoking during pregnancy on the risk of de novo and familial phacomatoses, respectively, was assessed using separate logistic regression models. Separate analyses were also conducted for neurofibromatosis, for the group “phacomatoses excluding neurofibromatosis”, tuberous sclerosis, Sturge–Weber syndrome, and for all other phacomatoses combined (i.e. excluding neurofibromatosis, tuberous sclerosis, and Sturge–Weber syndrome). Logistic regression models were adjusted for maternal and paternal age (categorized as <20, 20–24, 25–29, 30–34, 35–39, and 40+), birth year (categorized as 1982–1989, 1990–1999, 2000–2009, 2010–2014), and parity. Covariates were chosen a priori based on the previous literature, and their distribution was tabulated according to the type of phacomatosis and for the general population (Table 1). Data preparation and logistic regression analyses were performed using SAS software version 9.4. The study was approved by the Regional Ethical Review Board in Stockholm (dnr 2011/634–31/4, 2016/27–32, and 2018/1257–32).

**Table 1:** Descriptive characteristics and number of phacomatosis cases

	Individuals, n (%)	Phacomatoses de novo/familial	Neurofibromatosis de novo/familial	Phacomatoses excluding neurofibromatosis de novo/familial	Tuberous sclerosis de novo/familial	Sturge–Weber syndrome de novo/Familial	Other phacomatoses^a^ de novo/familial
**Maternal smoking during pregnancy**							
Non-smoker	2,633,407 (84.1)	1277/437	792/368	485/69	245/28	60/0	240/41
1–9 cig/day	325,059 (10.4)	157/61	105/54	52/7	24/5	8/0	20/2
10+ cig/day	173,590 (5.5)	112/30	60/26	52/4	24/0	7/0	21/4
**Parity**							
0	1,313,256 (41.9)	639/215	388/179	251/36	120/14	28/0	103/22
1	1,140,983 (36.4)	572/188	366/159	206/29	110/12	29/0	67/17
2+	677,817 (21.6)	335/125	203/110	132/15	63/7	18/0	51/8
**Maternal age**							
<20	64,147 (2.0)	37/13	23/13	14/0	6/0	2/0	6/0
20–24	546,122 (17.4)	248/112	142/97	106/15	47/3	15/0	44/12
25–29	1,057,238 (33.8)	516/202	316/173	200/29	86/15	30/0	84/14
30–34	954,191 (30.5)	486/137	297/112	189/25	105/10	17/0	67/15
35–39	426,069 (13.6)	210/56	144/47	66/9	39/5	11/0	16/4
40+	84,289 (2.7)	49/8	35/6	14/2	10/0	0/0	4/2
**Paternal age**							
<20	16,970 (0.5)	11/1	8/1	3/0	1/0	1/0	1/0
20–24	270,318 (8.6)	117/50	70/47	47/3	19/0	7/0	21/3
25–29	834,432 (26.6)	368/163	224/136	144/27	72/13	14/0	58/14
30–34	1,023,284 (32.7)	489/168	285/142	204/26	100/11	27/0	77/15
35–39	629,893 (20.1)	327/92	212/80	115/12	60/7	17/0	38/5
40+	357,159 (11.4)	234/54	158/42	76/12	41/2	9/0	26/10
**Birth year**							
1982–1989	699,945 (22.4)	392/114	255/99	137/15	66/4	14/0	57/11
1990–1999	979,550 (31.3)	555/156	337/123	218/33	118/11	23/0	77/22
2000–2009	925,109 (29.5)	460/180	287/152	173/28	83/14	23/0	67/14
2010–2015	527,452 (16.8)	139/78	78/74	61/4	26/4	15/0	20/0

**Note:** ^a^Excluding neurofibromatosis, tuberous sclerosis, and Sturge–Weber syndrome.

## Results

Among the 3,132,056 individuals included in the study, more than 2000 had a diagnosis of a phacomatosis (n=2 074). Descriptive characteristics of the included individuals and occurrence of phacomatoses are reported in Table 1. The most common phacomatosis subtype was neurofibromatosis (n=1 405), followed by tuberous sclerosis (n=326), and Sturge–Weber syndrome (n=75). Approximately 75% of all phacomatosis cases did not have a relative with a phacomatosis, i.e. were de novo (n=1 546). This proportion was lower for neurofibromatosis (68%, n=957), than for the group of phacomatoses excluding neurofibromatosis (88%, n=589). For tuberous sclerosis, it was 90% (n=293), while all cases of Sturge–Weber syndrome were de novo. Maternal smoking during pregnancy (light and heavy smoking combined) was more common during the first years of the study period: in 1982–1989 30% of the mothers reported smoking, and decreased to only 5% between 2010 and 2014 (data not shown). Estimated over the whole study period, 16% of the mothers were smokers at the first antenatal care visit.

Results regarding the effect of maternal smoking during pregnancy on the risk of de novo and familial phacomatoses are reported in Table 2. Fully adjusted analyses stratified according to familiarity showed an increased risk only for de novo phacomatoses (OR =1.21, 95% CI 0.99–1.48) associated with heavy maternal smoking (10+ cigarettes/day), while no effect was found for familial cases (OR =1.02, 95% CI 0.70–1.48) (Table 2). Analyses by subtypes of phacomatoses showed no association between maternal smoking during pregnancy (both light and heavy) and neurofibromatosis in offspring, regardless of familiarity (Table 2). The risk increase was confined to phacomatoses excluding neurofibromatosis; heavy maternal smoking during pregnancy was associated with an increased risk of de novo phacomatoses other than neurofibromatosis (OR =1.51, 95% CI 1.13–2.03), while no increased risk was indicated for familial cases. Similar findings were found when restricting the analysis to tuberous sclerosis, although confidence intervals were quite wide due to the limited number of cases (OR =1.39, 95% CI 0.91–2.14 for de novo tuberous sclerosis). Increased risk estimates associated with heavy maternal smoking were observed for de novo Sturge–Weber syndrome (OR =1.86, 95% CI 0.83–4.19) and de novo other phacomatoses combined (OR =1.57, 95% CI 0.99–2.50). Light maternal smoking during pregnancy was not associated with the occurrence of phacomatoses (Table 2).

**Table 2:** Effect of smoking during pregnancy on the risk of de novo and familial phacomatoses

	De novo cases		Familial cases	
N cases	Adjusted OR (95% CI)	N cases	Adjusted OR (95% CI)
**All phacomatoses**				
No smoker	1277	1.0 (ref)	437	1.0 (ref)
1–9 cig/day	157	0.93 (0.79–1.11)	61	1.09 (0.83–1.43)
10+ cig/day	112	1.21 (0.99–1.48)	30	1.02 (0.70–1.48)
**Neurofibromatosis**				
No smoker	792	1.0 (ref)	368	1.0 (ref)
1–9 cig/day	105	1.00 (0.81–1.23)	54	1.14 (0.85–1.53)
10+ cig/day	60	1.03 (0.79–1.35)	26	1.04 (0.69–1.56)
**Phacomatoses excluding neurofibromatosis**				
No smoker	485	1.0 (ref)	69	1.0 (ref)
1–9 cig/day	52	0.82 (0.62–1.10)	7	0.83 (0.38–1.82)
10+ cig/day	52	1.51 (1.13–2.03)	4	0.88 (0.32–2.45)
**Tuberous sclerosis**				
No smoker	245	1.0 (ref)	28	1.0 (ref)
1–9 cig/day	24	0.76 (0.50–1.17)	5	1.83 (0.69–4.84)
10+ cig/day	24	1.39 (0.91–2.14)	0	–
**Sturge–Weber syndrome**				
No smoker	60	1.0 (ref)	0	–
1–9 cig/day	8	1.11 (0.52–2.35)	0	–
10+ cig/day	7	1.86 (0.83–4.19)	0	–
**Other phacomatoses^a^**				
No smoker	180	1.0 (ref)	41	1.0 (ref)
1–9 cig/day	20	0.81 (0.51–1.30)	2	0.35 (0.08–1.44)
10+ cig/day	21	1.57 (0.99–2.50)	4	1.25 (0.44–3.57)

**Notes:** Adjusted for maternal age, paternal age, birth cohort, and parity.^ a^Excluding neurofibromatosis, tuberous sclerosis, and Sturge–Weber syndrome.

## Discussion

This is to the best of our knowledge the first study examining the effect of maternal smoking during pregnancy on the risk of de novo phacomatoses in offspring. While heavy maternal smoking was not associated with neurofibromatosis, an indication of an increased risk for de novo tuberous sclerosis was observed). Moreover, stronger associations were found for Sturge–Weber syndrome and all other phacomatoses combined, but only for heavy maternal smoking. No association between maternal smoking during pregnancy and familial phacomatoses was found, which supports the plausibility of the association with de novo phacomatoses.

Heavy maternal smoking during pregnancy was associated with an increased risk of phacomatoses in offspring for the group “phacomatoses excluding neurofibromatosis” which includes many phacomatosis subtypes, such as tuberous sclerosis, Sturge–Weber syndrome, and von Hippel–Lindau syndrome. Even though this is the largest study of phacomatoses to date, the number of cases of some of the specific types of phacomatoses was too small to allow statistically stable separate analyses. Indications of an increased risk were found for de novo tuberous sclerosis and Sturge–Weber syndrome, which suggest that maternal smoking during pregnancy might affect mutagenesis in TSC1, TSC2, and GNAQ. However, because of the limited number of cases of tuberous sclerosis and, especially, of Sturge–Weber syndrome (75 cases in total, of which only 8 exposed to heavy maternal smoking), results from these analyses should be interpreted with caution.

An increased risk was also observed for all other phacomatoses combined, i.e. after excluding neurofibromatosis, tuberous sclerosis, and Sturge–Weber syndrome. However, for the phacomatoses subtypes included in this group, which includes von-Hippel Lindau, Peutz-Jeger syndrome, and Cowden syndrome, the number of cases was too small to conduct separate analyses. Moreover, often the diagnosis reported to the health registers was simply “other specified phacomatosis” or “other unspecified phacomatosis”, which did not allow us to determine the phacomatosis subtype.

To the best of our knowledge, previous studies have not assessed or discussed the potentially harmful effect of smoking during pregnancy on the risk of de novo phacomatoses in the offspring or potential biological mechanisms: therefore, results from our study should be interpreted with caution and need to be replicated in other settings. Moreover, based on the modest increased risks observed in our study, only a small proportion of the cases of de novo phacomatoses can be explained by maternal smoking during pregnancy.

Neurofibromatosis is the most common phacomatosis subtype: over 65% of the phacomatosis cases had a diagnosis of neurofibromatosis in the current study. The large majority of de novo mutations causing neurofibromatosis are of paternal origin,^26^–^28^ thus, for this group no effect of maternal smoking during pregnancy is expected. However, in this study, which is based on register data, it was not possible to determine whether the de novo mutations that led to neurofibromatosis were of maternal or paternal origin: combining neurofibromatosis cases of maternal and paternal origin could have resulted in a dilution of the risk estimates for cases of maternal origin. Unfortunately, we did not have any information about paternal smoking at the time of conception, as the MBR contains information only on maternal smoking. Moreover, due to the coding practices for the register data used in the current study, we could not distinguish between neurofibromatosis type 1 and type 2, although other studies have reported that over 95% of the neurofibromatosis cases are neurofibromatosis type 1.^29^

The Multi-Generation Register was used to identify biological parents, siblings (full and half), grandparents, uncles, aunts, nephews, and cousins for all individuals with a phacomatosis, in order to determine whether at least one relative had a phacomatosis diagnosis reported in the Swedish health care registers. This information was the basis for the classification of cases as de novo or familial. There is a possibility that some cases were wrongly classified as de novo instead of familial, as the proportions of de novo cases reported by our study are higher than in previous studies.^5^,^7^ This discrepancy suggests that some relatives were not diagnosed or did not have their phacomatosis diagnosis reported in any of the Swedish health care registers. Possible reasons for the missing diagnoses can be variable expressivity with milder phenotypes that were not detected in relatives and the fact that the Inpatient Register became nationwide only in 1987 while the Outpatient Register was established only in 2001. Due to this potential disease misclassification, the effect of maternal smoking during pregnancy on the risk of de novo phacomatoses might have been underestimated. Thus, risk estimates reported in this study are likely to be conservative. However, the proportion of de novo cases in our study is in line with findings from a recent Dutch study that reported that approximately 74% of molecularly confirmed NF1 cases had de novo mutations.^9^ The fact that all cases of Sturge–Weber syndrome were de novo is not surprising, as this syndrome is not inherited but is caused by a somatic mosaic gene mutation that occurs during embryonic development.^10^ It is also possible that we were not able to identify all phacomatosis cases: however, this misclassification would have had an effect only on the precision of the risk estimates (i.e. the width of the confidence intervals) rather than on the validity of the findings.

Maternal smoking during pregnancy was recorded by the midwife during the first antenatal visit. This information referred to smoking during the first trimester which could differ from smoking status before or at the time of conception, as a pregnant woman could have quit smoking shortly after becoming pregnant. It is possible that some women may have under-reported their smoking due to social stigma against smoking during pregnancy. Importantly, the smoking information was recorded prospectively, i.e. prior to any knowledge about the pregnancy outcome, and therefore, misclassification of smoking status cannot give rise to spurious associations. The non-differential exposure misclassification could, however, have led to an underestimation of the effect of maternal smoking during pregnancy on the risk of phacomatoses in offspring.

Little is known about risk factors for de novo occurrence of phacomatoses. We adjusted all analyses for paternal age, which is the hitherto only known risk factor.^11^–^13^ We also adjusted for birth cohort to take temporal changes in smoking habits and health care data coverage into consideration. Parity was adjusted for because it could be related to maternal smoking during pregnancy and studies have found that it affects the risk of Down syndrome, even when adjusting for maternal age^30^ and therefore it might be implicated in the occurrence of genetic disorders in the offspring.

Our study indicates that maternal smoking during pregnancy might cause an increased risk of having offspring with de novo syndromes caused by errors that occurred during maternal meiosis II or later in the mosaic state during embryonic development. Modern massive parallel sequencing techniques have led to better knowledge about genetic mechanisms behind genetic diseases. In the current study, phacomatoses were classified according to the 8th, 9th, or 10th revisions of the ICD groups based on phenotypes alone. In the near future, it will be possible to perform more precise register-based studies in which patients can be divided into more exact disease subgroups based on genotype in combination with phenotype. In the current ICD classification, specific ICD codes are lacking for many rare diseases and there is, therefore, an urgent need for a better classification system, especially for phacomatoses in the “other phacomatoses” subgroup.

## Conclusion

In conclusion, this is the first study of maternal smoking during pregnancy and the risk of de novo phacomatoses. Although no association was observed for neurofibromatosis, heavy maternal smoking was associated with an increased risk of phacomatoses other than neurofibromatosis, including tuberous sclerosis and Sturge–Weber syndrome. The lack of association with familial phacomatoses strengthens the credibility of the associations. Further studies are needed to confirm our findings and elucidate potential biological mechanisms.
